# WHO Point Prevalence Survey to Describe the Use of Antimicrobials at a Tertiary Care Center in Pakistan: A Situation Analysis for Establishing an Antimicrobial Stewardship Program

**DOI:** 10.3390/antibiotics11111555

**Published:** 2022-11-04

**Authors:** Quratulain Shaikh, Samreen Sarfaraz, Anum Rahim, Aneela Hussain, Shameem Behram, Aamir Sikander Kazi, Mujahid Hussain, Naseem Salahuddin

**Affiliations:** 1 Infectious Diseases, Department of Medicine, The Indus Hospital and Health Network, Karachi 75190, Pakistan; 2 Indus Hospital Research Centre, The Indus Hospital and Health Network, Karachi 75190, Pakistan; 3 Department of Medicine, The Indus Hospital and Health Network, Karachi 75190, Pakistan; 4 Department of Pharmacy Services, The Indus Hospital and Health Network, Karachi 75190, Pakistan

**Keywords:** antimicrobial stewardship, drug resistance, survey, Pakistan, hospitalized

## Abstract

Antimicrobial stewardship is a systematic approach for promoting and monitoring responsible antimicrobial use globally. We conducted a prospective point prevalence survey of antimicrobial utilization among hospitalized adult patients during September 2021. The survey instrument was adapted from the WHO methodology for point prevalence surveys, and it was conducted at The Indus Hospital and Health Network, Karachi. Among the 300 admitted patients, 55% were males and the mean age was 44 (±18) years. At least 67% of the patients received one antimicrobial agent and the most common indication was surgical prophylaxis (40%). The most frequently used were antibacterial agents (97%) among all antimicrobials. Amoxicillin/Clavulanic acid and Ceftriaxone were the most frequently used antibacterial agents, i.e., 14% each. At least 56% of the antibacterial agents were amenable to antimicrobial stewardship when reviewed by infectious disease (ID) experts. Reasons for stewardship were: antibacterial not indicated (*n* = 39, 17.0%), unjustified prolonged duration of antibacterial (*n* = 32, 13.9%), extended surgical prophylaxis (*n* = 60, 26.2%), non-compliance to surgical prophylaxis guidelines (*n* = 30, 13.1%), and antibacterial not needed on discharge (*n* = 27, 11.7%). Median days of therapy (DOT) per agent was 3 days (IQR 2–4), while median DOT per patient was 2 days (IQR 1–4). These data have described the pattern of antimicrobial utilization in our institute. We found a higher prevalence of antimicrobial use overall as compared to the global figures, but similar to other low- and middle-income countries. Two important areas identified were the use of antimicrobials on discharge and extended surgical prophylaxis. As a result of these data, our institutional guidelines were updated, and surgical teams were educated. A post-intervention survey will help us to further determine the impact. We strongly recommend PPS at all major tertiary care hospitals in Pakistan for estimating antimicrobial utilization and identifying areas for stewardship interventions.

## 1. Background

Antimicrobial agents are a remarkable healthcare discovery of the 20th century [1,2]. A tremendous reduction in global mortality was achieved after the discovery of antimicrobials and improvement in public health practices. However, this achievement was soon overshadowed by the identification of antimicrobial resistance among many pathogens [3]. According to the World Health Organization, antimicrobial resistance (AMR) is a global health threat and requires an urgent multi-sectoral approach [4].

The WHO has defined antimicrobial stewardship (AS) as a coherent set of actions promoting the responsible use of antimicrobials across human health, animal health, and the environment, and at an individual, national, and global level [4]. In 1988, the Infectious Diseases Society of America (IDSA) issued a statement on the use of antimicrobial agents, thus introducing the concept of AS [5]. The World Health Assembly recognized the Global Action Plan on AMR in 2015, while the General Assembly declared AMR as a global threat in 2017 [4]. Pakistan developed its first National Action Plan for AMR in 2017 under the National Institute of Health (NIH) in accordance with WHO guidelines for the Global Antimicrobial Resistance and Use Surveillance System (GLASS). Currently, the AMR network has partnered with nearly 20 healthcare facilities all over Pakistan to establish a Pakistan AMR Surveillance System (PASS) [6].

The common contributing factors to the development of antimicrobial resistance are injudicious use of antibiotics, incorrect dosage, and duration [7,8,9]. Additional reasons cited in the literature include knowledge gaps among physicians prescribing antibiotics, unregulated pharmacy practices, availability of over-the-counter (OTC) antibiotics, lack of or poor regulations on antibiotic sales in the community, limited availability and cost of diagnostics, and last but not the least, poor literacy, enabling inappropriate use among consumers where prescription sharing and refills without consultation are common. Most of these reasons are applicable before a patient is admitted to a hospital, however, the major determinants of antibiotic utilization become the treating physician’s knowledge and implementation of an AS program’s recommendations once the patient is admitted to a healthcare facility [9,10,11,12,13,14,15]. However, there is no formal data on the existence and outcomes of antimicrobial stewardship programs in the country. Recent cross-sectional studies in the major hospitals of the largest province in the country (Punjab) showed rudimentary AS practices and at least 70% inappropriate antibiotic use among 4 hospitals in Lahore [13,15]. Another study in Lahore reported 90% prevalence of antibiotics in a large public sector hospital [16]. Structured AS programs exist in a mere handful of Pakistani hospitals. Judicious use of antibiotics, proper drug selection, and proper dosing during hospital admission are of immense importance in overall patient outcome and cost burden [17].

The Indus Hospital and Health Network (IHHN) is a nationwide network of at least 32 hospitals catering to a diverse patient population across the country. Interventions adopted at the main campus in Korangi, Karachi, are replicated and serve as roadmaps for other sites to follow. At the Indus Hospital, Korangi campus, we have a team of infectious disease (ID) specialists, infection prevention and control staff, microbiologists, and pharmacists who are working together for the development of an institutional antimicrobial stewardship program. One of the primary objectives of the AS program was to generate data on antimicrobial utilization at our campus. We conducted a prospective study among hospitalized patients to determine antibiotic utilization and prescribing patterns using a tool adapted from the WHO’s PPS [18].

## 2. Materials and Methods

We conducted a prevalence survey over 4 weeks in September 2021 including all admitted adult (≥18 years) patients at the Korangi Campus. The study was approved by the institutional IRB under the approval number IHHN_IRB_2021_08_001. A computer-generated electronic list of admitted patients was shared by the admissions department daily by 8 am starting from 1 September 2021. Patient profiles were reviewed and data were collected on the predesigned electronic form adapted from the WHO’s Point Prevalence Survey (PPS) instrument and designed on REDCap [12,17,18,19], hosted at the Indus Hospital and Health Network [20]. Patients who did not receive any antimicrobial were reviewed for demographic information only. Those who received an antimicrobial were evaluated in detail. The form included details on patient demographics, clinical presentation, initial diagnosis, use of empiric antibiotics, indications of antibiotic use, duration of antibiotic use, laboratory investigations for diagnosis of any infection, change of therapy to culture-directed antimicrobials, and the final outcome of the patient. If a prescribed antimicrobial was subject to stewardship, the reasons for stewardship were also determined. Trained infectious diseases (ID) physicians and one ID pharmacist were data collectors and none were directly involved in that patient’s care. Data on discharge medications and use of antimicrobials were also gathered. Data were analyzed using Stata version 14 [21]. Descriptive statistics are reported as frequencies and percentages for categorical variables. Quantitative data are reported as mean (SD) or median (IQR), as appropriate.

## 3. Results

A total of 300 patients were reviewed for the study admitted between 1 and 29 September 2021. The mean age of the patients (Table 1) was 44.3 (SD = 18.2) years. More than half (55.7%) of the patients were males. Most admissions (77%) were in the general ward and the most frequent admitting services were surgical specialties. There was no history of fever in 87% of the patients, while only 26% had a prior healthcare admission in the last 90 days. Only 27 (9%) of the patients were administered antibiotics in the ER. Only 9% required a central line insertion, while 98% had a peripheral vascular access during hospitalization. A urinary catheter was inserted in 37.9% of the patients. Fever was documented in 24.9% of the patients within 24 h of admission.

Among the 300 patients, 200 (66.7%) received at least 1 antimicrobial during their hospital stay (Table 2). Most (96%) of these were antibacterial. The most common indication for use of an antimicrobial was surgical prophylaxis (110/274, 40%), while the next most frequent was community-acquired infections (CAI) (77/274, 27%).

Half of the surgical prophylaxis in use was multiple dosing on more than one day (54%) and median duration of use was three days (IQR 2–4). For indications such as CAI or HAI, common etiologies were (Table 2) pneumonia (21%), gastrointestinal infections and skin and soft tissue infections (10%), and pyelonephritis (9%). However, in these cases, relevant culture samples were not drawn in 30% of cases, and hence culture-guided therapy was not possible. The most frequent antibiotics used were Amoxicillin/Clavulanic acid (*n* = 38, 13.8%), Ceftriaxone (*n* = 38, 13.8%), Cefuroxime (*n* = 31, 11.3%), Cefoperazone/sulbactum (*n* = 26, 9.5%), Meropenem (*n* = 21, 7.6%), Piperacillin/tazobactam (*n* = 21, 7.6%), and Metronidazole (*n* = 18, 6.5%). Approximately half of these antibiotics were administered prophylactically (*n* = 123, 44.4%).

Among the indicators of antimicrobial use (Table 3), it was observed that dosing and frequency were appropriate among 99% of agents. Among those with renal insufficiency, 96% had appropriate dose adjustment. Infectious disease consultation was sought on 37 (13.5%) patients, while implementation of ID recommendations occurred 86% of the time.

Among 244 antimicrobial agents used (Table 4), stewardship was applicable on 136 (55.7%). The most common reasons for application of stewardship were: antibiotics not indicated during hospitalization (*n* = 39, 17.0%), unjustified prolonged duration of antibiotics (*n* = 32, 13.9%), extended surgical prophylaxis (*n* = 60, 26.2%), non-compliance to surgical prophylaxis guidelines (*n* = 30, 13.1 %), and antibiotics not needed on discharge (*n* = 27, 11.7%).

A total of 167 microbiological specimens were sent (Table 5) to the laboratory, among which, 78/167 (46.7%) were blood cultures. The culture results were positive in 40/167 (23.9%). Most of the isolates were ESBL (*n* = 16, 41%), while only 3 were Carbapenem-resistant organisms.

In-hospital mortality was 2.7% (Table 6). Median days of therapy (DOT) per agent was 3 days (IQR 2–4). Among the patients who used antimicrobials, median days of therapy was 2 days (IQR 1–5). Among our patients, 78% (*n* = 153) received 1 antibiotic, 12% (*n* = 24) received 2 antibiotics, while 0.9% (*n* = 7) received 3 antibiotics during their hospital stay. Most patients (25%, *n* = 50) were on antimicrobials for 1 day, while 4% (*n* = 31) received them for 2 days, however the maximum duration of antimicrobial therapy was up to 56 days in 1 patient.

## 4. Discussion

Our results describe the most detailed data from Pakistan on antimicrobial use during hospitalization at a tertiary care center in the largest city on a standardized WHO PPS instrument. A recent review by Zikria et al. depicts that most PPS surveys are performed in high- and middle-income countries, with sparse data from low- and middle-income countries (LMIC). It is seen that our neighboring country, India, has the highest reported antimicrobial use [22]. The health system of Pakistan is very similar to that of India, but robust data from our country are lacking. Two recent papers from Pakistan show an alarming 89% and 77% point prevalence of antimicrobials in tertiary care hospitals in Punjab province, respectively [16,23]. In comparison, the Global PPS survey conducted among 53 countries worldwide reported an overall 34% use of antimicrobials among adult hospitalized patients, whereas that from East and South Asia combined was 37% [24].

In this study, antimicrobials were administered to half of all admitted patients and at least 50% among those could potentially be subject to stewardship, as reviewed by our experts. This is similar to global data, where approximately half of prescribed antimicrobials are deemed unnecessary [25]. The most common indication for prescribing an antibiotic was surgical prophylaxis, similar to a large European study, while community-acquired infections (CAIs) and healthcare-associated infections (HAIs) followed [26]. The most common HAIs were pneumonia and UTI, similar to previous local and global figures [22,27]. From a quality perspective, we saw poor (50%) documentation of indication for antimicrobial use, similar to a Ghanaian study, while a Canadian study and a European network had indication mentioned in 80% of the cases [28,29]. The Global-PPS which was conducted among 53 world countries showed that only 60% of antimicrobials in Asian hospitals have indications documented for use [24]. There was weak documentation of history of allergy (29%), in contrast to the western data where allergy records are complete in more than 97% of cases [30]. However, renal dosing was followed in nearly all (96%) patients in our study, in contrast to 67% inappropriate dosing from Zambia [22]. Parenteral routes were used to administer most of the antimicrobial agents, similar to the Global-PPS which reported at least 80% use of parenteral antimicrobial agents [24]. It was observed that more than 1/3 of the antimicrobials were never switched to oral in our survey, while a large European network study, ESAC-Net, also reported an only 4% switch to an oral antibacterial [26]. In cases where infectious disease advice was sought, it was followed in only 86%, which leaves at least 14% of the consultations being ignored and inappropriate antimicrobial use to continue.

In our data, it was seen that compliance to guidelines was only 41%, while in contrast, the Global-PPS survey on antimicrobial consumption revealed that guideline compliance was 76% overall. However, this may be due to the under-representation of LMICs (only 8 among 53 countries) where guideline-based practice is less robust compared to in the west due to reasons described later. We found that the top three antibiotics used were the beta-lactam group (Amoxicillin/Clavulanic acid), Ceftriaxone, and Cefoperazone/sulbactum, which is similar to figures reported by the Global-PPS survey [24]. Our institutional antibiogram reports 90% resistance to fluoroquinolones, hence the infrequent use of fluoroquinolones in our data.

The most common reasons for stewardship were prolonged surgical prophylaxis and non-compliance to surgical prophylaxis guidelines, as depicted in a recent Pakistani study including other global studies [16]. Poor compliance to guidelines either reflects a lack of knowledge among the physicians or a culture of injudicious antimicrobial use [13,31]. Results from our study were shared with the surgical specialties (orthopedics, general surgery, urology, and ENT) to highlight the role of the AS team and to emphasize the importance of following AS recommendations. Similar behavioral interventions have proven to be successful in curtailing inappropriate antimicrobial utilization [32].

A major area for stewardship often overlooked by physicians is the over-prescription of antibacterial agents on discharge, as highlighted by Vaughn in his recent review article [33]. These authors identified that 1 in 8 patients are given an antibacterial on discharge and approximately half of them are avoidable. Research shows that most physicians in LMICs, even at large multidisciplinary centers, lack adequate knowledge on the problem of growing AMR and rely heavily on antimicrobial use for minor viral ailments [34]. Determinants of antimicrobial overuse by physicians include a lack of basic knowledge on appropriate antimicrobial use and pressure from cultural norms or patients’ expectations to administer antibiotics [35]. Many studies globally report overuse of antibiotics on discharge, even in well-developed healthcare systems in the world [36,37,38]. This leads to increased cost, unwanted side effects, including *Clostridioides difficile* infections, which can be serious, and increased incidence of antimicrobial resistance. We found that 38% of our patients received an antibiotic on discharge, among which at least 11% were not needed. Multiple targeted interventions to curtail the use of antimicrobials on discharge have been proposed and are currently being tested and reported [38].

An important area of debate between infectious disease experts and surgeons has remained that of extended surgical prophylaxis. Although some data from observational studies [39] recommend fewer surgical site infections when administered extended prophylaxis beyond the standard 24 h, a recent meta-analysis published in The Lancet Infectious Diseases has clearly shown that there is no benefit to extended surgical prophylaxis in most patients, except a small proportion who did not receive timely preoperative prophylaxis or did not get repeat antibiotic dosing in prolonged surgeries [40]. In light of these findings, we revised our surgical prophylaxis guidelines to further emphasize the role of standard prophylaxis lasting up to 24 h and no more.

Although we acknowledge that our study period is short, it was resourcefully exhaustive to prospectively review consecutive admissions any further. We believe our study’s aim for this point prevalence survey was fulfilled through this sample size. A possible limitation of our data is the large proportion of surgical patients, however this, in itself, facilitated the identification of a core area for AS intervention, i.e., extended surgical prophylaxis. Thirdly, since each case was reviewed by one ID physician, the decision on whether the use of antimicrobial was justified or not may be subjective, however, through standardization of the instrument and increasing objectivity through reasons for injudicious use, this subjectivity was minimized. If resources allowed, we would adjudicate each case by at least 2 ID physicians to reduce subjectivity. We acknowledge that some of the data were missing, which has been explicitly described in the tables. Due to the lengthiness of the survey, it was an expected situation, but we have made sure that it remains below 5% for our main outcome measures.

Our study represents a point prevalence survey of antibiotic utilization among patients admitted to one of the leading hospitals in the largest city of Pakistan. We have identified areas for targeted intervention to reduce antimicrobial consumption at our institute. We aim to complete a follow-up survey for measuring the impact in the different areas where interventions are being planned. It is important to measure the use of antimicrobials at other hospitals within the country to develop a situation analysis, after which a framework of interventions can be designed. The problem of antimicrobial resistance is a global problem which needs better recognition and urgent handling.

## Figures and Tables

**Table 1:** Demographic and clinical details of participants at admission.

	*N* = 300 (%)
**Age** years, mean ± SD	44.3 ± 18.2
**Gender**	
Male	167 (55.7)
**Mode of Admission**	
Emergency Room	146 (48.6)
Elective Admission	150 (50.0)
Unknown	4 (1.3)
**Admitting Ward**	
General ward	228 (77.0)
HDU—High dependency unit	28 (9.5)
MICU—Medical ICU	4 (1.4)
SICU—Surgical ICU	3 (1.0)
CCU—Coronary Care Unit	33 (11.1)
Unknown	6 (0.02)
**Admitting Service**	
Orthopedics	49 (16.3)
Urology	44 (14.6)
General Surgery	40 (13.3)
Cardiology	38 (12.6)
Internal Medicine	29 (9.6)
Infectious Disease	22 (7.3)
Gastroenterology	21 (7.0)
E.N.T. (Ear, Nose, Throat)	17 (5.6)
Nephrology	16 (5.3)
Pulmonology	12 (4.0)
Hematology	4 (1.3)
Plastic surgery	2 (0.6)
Unknown	6 (0.02)
**Febrile at admission**	
Yes	24 (12.3)
**Admission to HC facility in past 90 days**	
No	131 (43.6)
Yes	50 (16.6)
Unknown	119 (39.6)
**Use of antimicrobial by patient before admission**	
No	218 (72.6)
Yes	25 (8.3)
Unknown	57 (19.0)
**Antibiotic Administered in ER**	
No	118 (41.0)
Yes	27 (9.4)
Not applicable (not admitted through ER)	132 (45.8)
**Central vascular catheter during admission**	
No	163 (54.3)
Yes	18 (0.06)
Unknown	119 (39.6)
**Peripheral vascular catheter during admission**	
Yes	193 (98.5)
**Urinary catheter during admission**	
No	103 (34.3)
Yes	74 (24.6)
Unknown	123 (41.0)
**Intubation during admission**	
Yes	73 (37.2)
**Stay in critical care area**	
Yes	28 (14.4)
**TLC count on admission** × 10^9^ (mean ± SD)	10,147 ± 5364
**Fever within 24 h of admission (Yes)**	48 (24.9)
**History of allergy recorded**	
No	56 (18.6)
Yes	136 (45.3)
Unknown	108 (64.0)

HC = healthcare; ER = emergency room; TLC = total leucocyte count.

**Table 2:** Use and pattern of antimicrobial use.

	*N* (%)
**Antimicrobial use during hospital stay**	200 (66.7)
Antibacterial	198 (96.6)
Antifungal	3 (1.5)
Antimalarial	4 (2.0)
**Indication mentioned for antibiotic use (Yes)**	127 (63.5)
**Indication type, *n* = 274**	
Healthcare-associated infections—HAI	62 (22.6)
Community-associated infections—CAI	77 (28.10)
Surgical Prophylaxis	110 (40.1)
Medical Prophylaxis	21 (7.6)
Other	4 (1.4)
**If surgical prophylaxis, then duration, *n* = 110**	
One dose	10 (10.0)
Multiple doses on one day	40 (36.4)
Multiple doses on more than one day	60 (54.0)
**Duration of extended surgical prophylaxis^@^, *n* = 52**	
Median (IQR) days	3.0 (2.0–4.0)
Minimum–maximum days	1–14
**Site of surgical prophylaxis, *n* = 110**	
Otolaryngology	8 (2.8)
Cardiovascular	2 (0.7)
Gastrointestinal	22 (7.8)
Skin soft tissue bone and joint	37 (13.2)
Urinary tract	37 (13.2)
Unknown	4 (1.4)
**HAI/CAI Site of Infection ^¥^, *n* = 139**	
Infections of the central nervous system	2 (1.5)
Infections of ear, nose, throat, larynx, and mouth	11 (8.4)
Pneumonia	28 (21.2)
Gastrointestinal infections	13 (9.9)
Intra-abdominal sepsis, including hepatobiliary	3 (2.3)
Surgical site infection involving skin or soft tissue but not bone	13 (9.9)
Cellulitis, wound, deep soft tissue not involving bone, not related to surgery	5 (3.8)
Septic arthritis, osteomyelitis of surgical site	2 (1.5)
Septic arthritis, osteomyelitis, not related to surgery	1 (0.8)
Symptomatic lower urinary tract infection (e.g., cystitis)	7 (5.3)
Symptomatic upper urinary tract infection (e.g., pyelonephritis)	12 (9.2)
Asymptomatic bacteriuria	4 (3.1)
Laboratory-confirmed bacteremia	9 (6.9)
Clinical sepsis	9 (6.9)
Febrile neutropenia or other form of manifestation of infection in immunocompromised host	3 (2.3)
Systemic inflammatory response with no clear anatomical site	5 (3.8)
Completely undefined, site with no systemic inflammation	5 (3.7)
Missing	7 (0.05)
**Relevant culture taken before starting antibiotics, *n* = 274**	
Partially	18 (6.5)
Yes	102 (37.2)
No	82 (29.9)
Not Applicable (e.g., used as prophylaxis)	69 (25.1)
Unknown	3 (1.0)
**Antibiotics used, *n* = 274**	
Amikacin	13 (4.7)
Amoxicillin/Clavulanic acid	37 (13.5)
Azithromycin	4 (1.5)
Benzathine benzyl penicillin	1 (0.4)
Cefazolin	11 (4.0)
Cefoperazone/sulbactum	26 (9.5)
Ceftazidime	1 (0.4)
Ceftriaxone	38 (13.8)
Cefuroxime	31 (11.3)
Ciprofloxacin	14 (5.1)
Clarithromycin	1 (0.4)
Clindamycin	3 (1.1)
Colistin	3 (1.1)
Ertapenem	1 (0.4)
Fosfomycin (oral)	1 (0.4)
Levofloxacin	4 (1.5)
Meropenem	21 (7.6)
Metronidazole	18 (6.5)
Piperacillin/tazobactam	21 (7.6)
Rifaximin	2 (0.7)
Sulfamethoxazole/trimethoprim	2 (0.7)
Tigecycline	1 (0.4)
Vancomycin	11 (4.0)
Other *	9 (3.2)
**Route of administration**	
Oral	45 (16.4)
Parenteral	229 (83.5)
**If Parenteral, Type**	
Intramuscular	1 (0.4)
Intravenous intermittent	219 (79.9)
Intravenous continuous infusion	1 (0.4)
Other	11 (4.0)
Unknown	42 (15.3)
**If parenteral, oral switch done, *n* = 229**	
No	100 (43.6)
Yes	36 (15.7)
Unknown	93 (43.6)
**No. of missed Doses**	0
**Treatment Type, *n* = 274**	
Directed	36 (13.1)
Empiric	107 (39.0)
Prophylaxis (surgical + medical)	131 (47.8)

* 2 Voriconazole, 1 Isoniazid, 1 Ethambutol, 1 Fluconazole, 1 Rifampicin, 1 combination Anti-tuberculous drugs, 1 Nitazoxanide. ^¥^ Classification based on WHO PPS. **^@^** Surgical prophylaxis beyond the recommended duration in institutional guidelines, generally more than 24 h.

**Table 3:** Antimicrobial use indicators.

	*N* (%)
**Guideline compliance**	
No	105 (38.0)
Yes	114 (41.6)
Not assessable (when more than 1 indication)	19 (6.9)
No information	36 (13.1)
**Unit dose of antimicrobial**	
Appropriate	269 (98.1)
Inappropriate	4 (1.4)
Unknown	1 (0.3)
**Frequency of antimicrobial**	
Appropriate	269 (98.2)
Inappropriate	1 (0.4)
Unknown	4 (1.4)
**Empiric therapy correctly modified according to culture susceptibility (*n* = 107)**	
No	59 (55.1)
Yes	24 (22.4)
Unknown	24 (22.4)
**Empiric therapy discontinued within 5 antibiotic days due to lack of culture reports (*n* = 63)**	
No	42 (66.6)
Yes	19 (30.1)
Unknown	2 (3.1)
**Dose and dosing interval adapted to renal function**	
No	4 (3.5)
Yes	110 (96.5)
**ID consultation (*n* = 274)**	
No	210 (76.6)
Yes	37 (13.5)
Unknown	27 (9.8)
**Compliance to ID recommendation (*n* = 36)**	
No	5 (13.8)
Yes	31 (86.1)
Unknown	1 (2.7)

**Table 4:** Antibiotic stewardship.

	*N* (%)
**Antimicrobial stewardship (*n* = 274)**	
Not applicable	108 (44.3)
Applicable	136 (55.7)
Missing	30 (10.9)
**Reasons for antimicrobial stewardship**	
Antibiotic not indicated	39 (17.0)
Unjustified prolonged duration of therapy	32 (13.9)
Non-compliance to surgical prophylaxis guidelines	30 (13.1)
Extended surgical prophylaxis	60 (26.2)
Antibiotic on discharge not needed	27 (11.7)
Microorganism resistant to antibiotic used	6 (2.6)
Inappropriate choice of antibiotic	11 (4.8)
Restricted antibiotics use	5 (2.1)
Narrow spectrum options available	5 (2.1)
Incorrect dose	4 (1.7)
Contraindication to use of current antibiotic	2 (0.8)
Overlapping spectrum	2 (0.8)
Other	6 (2.6)

**Table 5:** Microbiological data.

Specimen Type (*n* = 167)	*N* (%)
Blood	78 (46.7)
Urine	57 (34.1)
Sputum/Respiratory Sample	6 (3.6)
Wound	7 (4.2)
Sterile Fluids	3 (1.8)
Other	16 (9.6)
**Culture Results (*n* = 181)**	
CS not sent	16 (8.8)
Positive	40 (22.1)
Negative	125 (69.1)
**Resistant Phenotype (*n* = 32) ^¥^**	
Methicillin-resistant *Staphylococcus Aureus*	2 (5.1)
Vancomycin-resistant enterococcus	1 (2.6)
Carbapenem-resistant Gram-negative rod	3 (7.7)
Third-generation Cephalosporin-resistant Gram-negative rod	16 (41.0)
Not resistant	10 (25.6)

^¥^ Based on phenotypic drug sensitivity testing.

**Table 6:** Outcomes.

Final Status of the Patient	*N* (%)
Alive	289 (96.3)
Dead	8 (2.6)
Unknown	3 (0.01)
**Antimicrobials on Discharge**	
Yes	115 (38.3)
DOT * per antibacterial, *n* = 286	
Median (IQR) days	3.0 (2.0–4.0)
Minimum–maximum days	1–7
DOT * per patient, *n* = 195	
Median (IQR) days	2.0 (1.0–5.0)
Minimum–maximum days	1–56
**Number of antimicrobials per patient**	
Median (IQR)	1 (1–1)
Minimum–maximum	1–8

* Days of therapy.
